# The roots of the concept of depth art

**DOI:** 10.1017/S2045796024000398

**Published:** 2024-10-08

**Authors:** Goran Stojčetović

**Affiliations:** Independent Scholar, Serbia

I spent my childhood and youth days in the town of Uroševac, where I lived with my parents and brother, and this period of my life was full of ambivalence. On the one hand, my parents noticed early and supported my interests in music, drawing and mathematics, and on the other hand, the extreme social and political situation around us profoundly influenced my youthful personality and forced me to rebel against authority and accepted social values. After finishing elementary and secondary education with excellent grades, even receiving municipal and regional awards in art and mathematics, and playing music in a band, more and more I gave into drinking, street fights and troubles with the police. After finishing high school, I enrolled at the Faculty of Arts in Priština where I studied painting. However, during my third year of studies, I failed to get a passing grade in painting because, according to my professor, I ignored her instructions, I didn’t come to all her classes and was untalented. I was forced to repeat a year and continue my painting classes with a different professor, which was very stressful. During 1997, I fanatically started doing drawings with a ballpoint pen, which were filled with anxiety and frustration. I had a vague desire to draw the most disgusting motifs and later put them into one bizarre, terrifying whole. Such mental state was accompanied by psychosomatic reactions which manifested in the form of morning sickness and vomiting. More and more I retreated from the social life. Luckily, during the same period, I had several opportunities to talk with a friend of my father’s, who was a neuropsychiatrist, and who introduced me to a psychological analysis of artistic expression by interpreting my drawings. His Jungian way of deeper observation of visual expression and symbolism utterly changed my relationship towards life and art.

In hindsight, it seems quite natural that these big changes of mine occurred at the same time the changes in a world around me began to unravel. I believe it hugely important for the understanding of my work to recount all the states I lived in, which appeared and disappeared in a very short period of history. So, since I was born in 1975 until 1999, I lived in Kosovo and Metohija, the Autonomous Province of the Republic of Serbia within the Socialist Federal Republic of Yugoslavia (1945–1992), followed by the Federal Republic of Yugoslavia (1992–2003), then Serbia and Montenegro (2003–2006), and, ultimately, since 2006, the Republic of Serbia, where I live today. The collapse of the Socialist Federal Republic of Yugoslavia (SFRY)brought about a bloody civil war, mass graves, hyperinflation, increase in crime and addiction, international sanctions and, eventually, in 1999, the bombing of my country by the NATO alliance. The three months of bombing I spent with my family and neighbours in the cellar of a residential building in Uroševac, where I mostly drew human heads, anthropomorphic animals and monsters, usually on pages of small political party promotional notebooks. Each drawing was made without any thinking or conscious intent.

And so it was until the day I had a specific experience, which I recorded directly through drawing, and which, I believe, created a strange conditioned reflex, one that would awake a psychosomatic need to actively seek external sources of intense sensory stimuli. I was awakened from a dream by the intense sound, which I thought was the sound of a missile striking the roof of our building, but luckily for me, the explosion took place a couple of kilometres away. People around me were fleeing in panic, while I calmly sat down on the bed and started to draw. I can’t remember anything after that, but the drawing revealed the head of a man whose brain had slid down his neck, except for the swollen and twisted veins of his nervous system, that were somehow still attached to the hollowed-out skull, creating the impression of the Gorgon Medusa. The eyes were in a state of hypnotic shock and looking to the side. I used blue and red ballpoint pens on a small piece of paper, which was the same dimensions as the palm of my hand, which served as a drawing desk. In this way, I created something very much alive, intimate and mobile. Something I could keep firmly in my grasp and treasure like it was the last drop of water.

The signing of the Kumanovo Agreement brought about the end of the bombing, but, in a wave of violence that ensued, perpetrated by Albanian nationalist extremists, I was forced to leave my home. Together with my family and the entire Serbian population of Uroševac I left Kosovo and Metohija on 17 June 1999, and spent my refugee days first in the city of Niš, and later in the village of Rogača, near Belgrade. In the meantime, I graduated from the Priština Faculty of Arts. Paradoxically, I remember this refugee period as the most fruitful in my life because I was free to start working on a complete spiritual reintegration, deep in the countryside and isolated from any media. From early morning until midnight, almost every day, for two years, I just drew, painted, read and wrote down my thoughts in a diary I called Between Work and Sleep. I studied passionately the works of Carl Gustav Jung and theoretical works of artist Paul Klee. Inspired by Klee’s geometric analyses of artistic expression, I began my own geometric research, often consulting with my father, who was a professor of mathematics. On the other hand, I fanatically drew whenever I felt anxious or angry, or had a panic attack, or was frightened by oppressive thoughts or apocalyptic visions of the world, or experiencing messianic fervour, and I would do it just about anywhere, anytime, with anybody, on any surface, in any kind of environment and state. The uninterrupted speed of movements, which the ballpoint pen provided me with, accelerated my own sense of liberation, but also invoked some kind of normalization, since I started to perceive key elements of my unstable side of personality. Also, I was completely fascinated by the characteristic indigo blue tone of the ink inside the ballpoint pen. In time, a series of meanings and symbols related to this colour began to emerge. From the fact that deep blue was actually the first light that hit you after darkness, or that it was the colour of plums, a fruit the Serbs consider their national treasure, since they use it to make another national product, the famous Slivovitz plum brandy, or that it was the colour of police force, or the colour of worker’s clothes in socialist Yugoslavia, and, finally, the colour of bruises. It was also fascinating to me watching on TV how the then president Slobodan Milošević was repeatedly drawing his personal blue line on paper with a ballpoint pen at various peace conferences, while simultaneously turning me and hundreds of thousands of people into refugees and statistical numbers.

**Figure 1.**
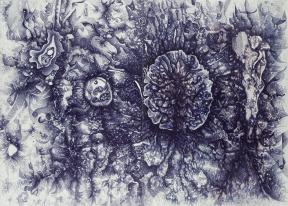
Goran Stojčetović, *Big Blue 2*, 2009, ballpoint pen on paper, 100 × 140 cm.

**Figure 2.**
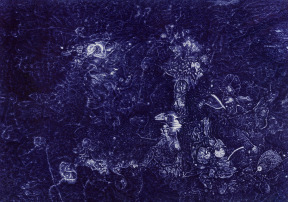
Goran Stojčetović, *Big Blue 3*, 2009, ballpoint pen on paper, 70 × 100 cm.

**Figure 3.**
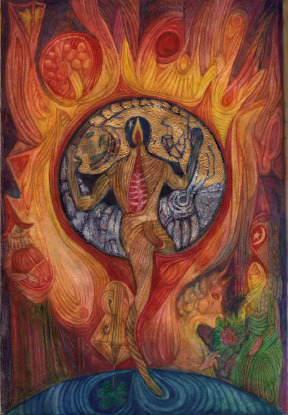
Goran Stojčetović, *Anthropos Autopsis* (the Book of Visions), page 17, 2022, watercolour on paper, 30 × 20.5 cm.

At the same time, I felt the need to talk about art with as many people as possible. Later, these conversations evolved into some sort of get-togethers where I drew side by side with different groups of people, or worked individually, but also collaborated on each other’s work. It was then, in 2006, and based on these experiences, that I first wrote down and sketched a vision I had, about the existence of a creative system, which I then called The Seven Circles of a Spiral, but after a few years I renamed it Depth Associative Drawing, or simply, *Depth Drawing*. There, in broad strokes, and in 7 Points, I described the phenomena that kept reappearing and felt interconnected. For example, I noticed that no matter how fast or spontaneous the first movement I applied to any surface was, it was actually repeatedly determined by the unconscious feeling that there was some personal, unique point of pre-ordained position, the feeling of your own coordinates within the general system of space and time. Thus, I began to record my impressions on all my psycho-physical characteristics emerging immediately before, during and after I would conclude a creative act. Everything was important to me, from where on the surface I usually began, what was the intensity, the speed and duration of movements, to what was the form, state and the contents of all those free associations, which came to me during sudden exposure to unarticulated visual content. Simply put, the relationship ‘with myself, within myself, inside any given moment of space and time’ was the only measure of worth I drew inspiration from.

**Figure 4.**
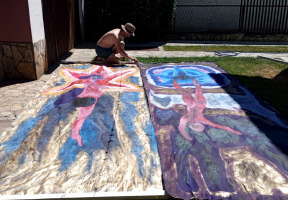
Goran Stojčetović working on a large format painting, 2023, 450 × 320 cm.

At the same time, I discovered the studies of Leopold Szondi on the ‘family unconscious’. His Fate Analysis proved the existence of a genetic inheritance within each of us that unconsciously determined various choices in life, from our loved ones, friends, ideals, professions, illness and death. An insight into the complexity of the very notion of choice, which preceded any action and expression, developed in me a somewhat ‘mysterious’ ability to see through drawing problematic personality mechanisms or even concrete traumatic experiences of people I knew nothing about. Thus, I realized I had to face my own problem of alcohol addiction and that this was *conditio sine qua non* in my becoming an artist. Years passed, until January 2013, when, after two days of heavy drinking, I decided to leave my family and civilian life, and move to my village on the slopes of Šar Mountain in Kosovo, where I would dedicate myself exclusively to art. After I woke up drunk one night in Kosovska Mitrovica, I experienced a profound vision. I saw a message, in the form of a TV headline, which read: ‘Goran Stojčetović does not exist anymore. Only art brut, no more running away’. I immediately realized I was through with alcohol and very soon I founded the Art Brut Serbia group, and a year after that, an official association bearing the same name. It provoked an interest in many journalists, psychologists, artists and other cultural workers, and, most importantly, unknown creators, who contacted me from all across the country and who came from various margins of society. More and more, it became clear to me that, in my country, there was not a single social platform that could provide people with the possibility to express themselves creatively or, in any way, channel all the repressed feelings and experiences from recent wars and bombing. I began a project which focused on the collecting, analysis and exhibiting the works of untrained, or ‘non-academic’ artists made during the NATO bombing; I founded the Art Brut Studio at the Psychiatric Clinic of the Military Medical Hospital in Belgrade, where I still work regularly with patients, many of whom are soldiers and other military personnel, who experienced war trauma directly or indirectly; I organized workshops that dealt with issues of addiction and suicide; I drew together with prisoners, children without parental care, young people with intellectual disabilities, Serbian and Roma youth living in ghettos, civilians and The Kosovo Forcesoldiers in Kosovo.

And so it was until the start of the Covid-19 epidemic, when a period of isolation brought me back to myself and faced me with all the contents and the accumulated experience of drawing together with roughly 2000 people. Then I experienced several visions that determined the future direction of my artistic activity. From the beginning of 2020 to 2023, I created a whole system of interconnected art forms, completely inspired by visions. I will mention Anthropos Autopsis, the book of visions in watercolours technique; the drawings made with wooden colour pencils from the series The Diary of Black Pygmalion; photographs from the Catacomb Theater series, and the latest series of large-format paintings made with acrylic on cardboard and oilcloth.

Somehow, all these circumstances freed me from all my other addictions, namely, anxiolytics and nicotine, and now I am even more dedicated to exploring the concept of *depth art* as art of the inner being of each individual on this planet.

